# 

**Published:** 1917-07

**Authors:** 


### ANNOUNCEMENTS

---

THE AMERICAN SOCIETY OF ORTHODONTISTS. The next meeting of this Society will be held in Excelsior Springs, Missouri, September 5-8. An attractive program is being prepared and it is hoped there will be a large attendance. For further information, address Dr. F. M. Casto, Rose Building, Cleveland, Ohio.

---

NATIONAL DENTAL ASSOCIATION. As already announced the annual meeting will be held this year in New York City, at the Astor House, October 22 to 26. For particulars, address the Secretary, Dr. Otto U. King, Huntington, Indiana.

---

NATIONAL ASSOCIATION OF DENTAL FACULTIES will hold its annual meeting in New York at the Astor House, October 19 and 20. Dr. C. C. Allen, Secretary, Kansas City, Missouri.

ROCHESTER DENTAL DISPENSARY. The graduating exercises for the first class of New York Oral Hygienists was held in the new dispensary building, June 14, 1917. Thirty-eight women were graduated, after a course of eight months of study and preparation. All the graduates have positions, either in offices or as inspectors or workers in schools or public institutions.

---

### NATIONAL DENTAL ASSOCIATION ANNOUNCEMENTS

### Partial Program

Up to date the following reports have been received from the various Section Chairmen, Committeemen, etc.:

Section I. Chairman, Dr. E. D. Coolidge, 59 East Madison Street, Chicago, Illinois.

"Some Neglected Operative Prerequisites," by Dr. Fred E. Hart, of San Francisco, California.

"Porcelain Inlays" (except title not yet chosen), by Dr. W. L. Fickes, Pittsburgh, Pennsylvania.

"Interpretation of Radiographs," by Dr. Howard R. Raper, of Indianapolis, Indiana.

"Present Tendencies in Operative Dentistry," by Dr. J. M. Walls, of St. Paul, Minnesota.

Also an important paper dealing with the subject of dental education. Essayist not selected as yet.

Section II. Chairman, Dr. F. B. Moorehead, People's Gas Building, Chicago, Illinois.

Dr. Virgil Loeb of this committee reports that he has thus far accepted two essayists. Dr. Elmer S. Best, of Minneapolis, on some phase of root canal filling; and Dr. Howard R. Raper, of Indianapolis, on "Misinterpretation of Radiographs."

Section III. Chairman, Dr. L. E. Custer, 28 North Ludlow Street, Dayton, Ohio.

"Ionization. With Special Reference to Ionic Chemistry," by Dr. Geo. T. Fette, Cincinnati, Ohio.

"The Chemical Action of Soil Bacteria on Calcium Phosphates, with the Chemical Analysis of the Human Teeth," by Dr. J. E. Hinkins, of Chicago, Illinois.

"Why Measurements of the Mandible, Tracings of the Condyles, the Construction of Hypothetical Triangles, and the Use of the Face-Bow, are all Nonessential in the Construction of Dentures Possessing the Highest Degree of Efficiency," by Dr. D. D. Campbell, of Kansas City, Missouri.

Paper, subject to be announced later, by Dr. Calvin S. Case, of Chicago, Illinois.

Also two other papers, titles of which will reported later.

STATE SOCIETY OFFICER'S SECTION. Chairman, Dr. John C. Forsyth, 430 East State Street, Trenton, New Jersey.

First session. "Some Phases of Post-Graduate Work," by Dr. B. L. Shobe, Tulsa, Oklahoma.

"Securing Some Satisfactory Legislation," by Dr. Alexander H. Reynolds, Philadelphia, Pennsylvania.

The second session will be devoted to six or seven short papers of five to ten minutes each by men of different state societies, telling of some outstanding feature of their state society's work that is thought to be of the greatest importance, or, if the essayist prefers, he may present the weak part of the work and ask for suggestions to help them out. These papers are to be followed by a general discussion which we hope will bring out some very valuable points. The means of the essayists for this session have not as yet been secured.

COMMITTEE ON ANESTHETICS. The secretary of this committee, Dr. Chalmers J. Lyons, of Ann Arbor, Michigan, reports as follows:

"The Teaching of Conductive Anesthesia," by Dr. Theodore Blum, of New York City.

"Afterpain in Local and General Anesthesia," by Dr. A. E. Hertzler, of Kansas City, Missouri.

"The Toxicity of Local Anesthetics," by Dr. Geo. B. Roth, of Washington, D. C.

COMMITTEE ON ENTERTAINMENT. At a recent meeting of this committee a Ladies' Auxiliary was organized, of which Mrs. Dr. M. L. Rhein was made the chief officer, and Mrs. Dr. Henry W. Gillett, 140 West 57th Street, New York City, Secretary. It would greatly facilitate the endeavors of the Ladies' Auxiliary to add as much as possible to the pleasures and comforts of the visiting ladies, if those who intend to come to New York would notify Mrs. Gillett, stating if possible, the hotel at which they will be registered. R. OTTOLENGUI,

*Chairman Publicity Committee.*

---

### SALIVA CONTROL

I frequently get a patient who the moment he opens his mouth, the saliva rolls out and it's the devil to do anything in that mouth. Is this your own experience?

Of course, to give your patient a dose of atropin sulphate before the operation to dry the mouth is nothing new, but this is not always practical.

Not long ago I got an inspiration; how I got it is a mystery. I was working for one of these impossible cases where the dam was not practical, when this inspiration came to me. I took a little sample can of corega which had been sent me, and sprinkled some of the powder upon a small dampened napkin, folded it, dried the inside of the cheek and slapped this pad (?) up against it and held it there with a cotton roll, and believe me, I got through the operation without one drop of saliva coming from the duct of Steno, or any part of the surface of the cheek covered by the pad!

Since then, this has been part of my everyday procedure. I never have had very much trouble controlling the flow from the sublingual glands, but it's the cheeks which have always worried me; and now that worry's over. If you have had this trouble in the past, try this out.—C. Edmund Kells, in *Oral Hygiene.*

